# Care of the mother-infant dyad: a novel approach to conducting and evaluating neonatal resuscitation simulation training in Bihar, India

**DOI:** 10.1186/s12884-017-1434-1

**Published:** 2017-07-28

**Authors:** Brennan Vail, Hilary Spindler, Melissa C. Morgan, Susanna R. Cohen, Amelia Christmas, Pramod Sah, Malay B. Shah, Aritra Das, Dilys M. Walker

**Affiliations:** 1 Department of Pediatrics, University of California San Francisco, 550 16th Street, 4th Floor, 4551, Box 0110, San Francisco, CA 94143 USA; 2 Global Health Sciences, University of California San Francisco, 550 16th St, San Francisco, CA 94158 USA; 3 Department of Pediatrics, University of California San Francisco, 550 16th St, Box 1224, San Francisco, CA 94158 USA; 4 College of Nursing, University of Utah, 10 South 2000 East, Salt Lake City, UT 84112 USA; 5 Pronto International, State RMNCH+A Unit, C-16 Krishi Nagar, A.G. Colony, Patna, Bihar 80002 India; 6 CARE India, 14 Patliputra Colony, Patna, Bihar 800013 India; 7 Department of Obstetrics and Gynecology and Reproductive Services, University of California San Francisco, 1001 Potrero Ave, San Francisco, CA 94110 USA

**Keywords:** Early neonatal mortality, Neonatal resuscitation, Simulation training, Mother-infant dyad

## Abstract

**Background:**

As the global under-five mortality rate declines, an increasing percentage is attributable to early neonatal mortality. A quarter of early neonatal deaths are due to perinatal asphyxia. However, neonatal resuscitation (NR) simulation training in low-resource settings, where the majority of neonatal deaths occur, has achieved variable success. In Bihar, India, the poorest region in South Asia, there is tremendous need for a new approach to reducing neonatal morality.

**Methods:**

This analysis aims to assess the impact of a novel in-situ simulation training program, developed by PRONTO International and implemented in collaboration with CARE India, on NR skills of nurses in Bihar. Skills were evaluated by clinical complexity of the simulated scenario, which ranged from level 1, requiring NR without a maternal complication, to level 3, requiring simultaneous management of neonatal and maternal complications. A total of 658 nurses at 80 facilities received training 1 week per month for 8 months. Simulations were video-recorded and coded for pre-defined clinical skills using Studiocode™.

**Results:**

A total of 298 NR simulations were analyzed. As simulation complexity increased, the percentage of simulations in which nurses completed key steps of NR did not change, even with only 1–2 providers in the simulation. This suggests that with PRONTO training, nurses were able to maintain key skills despite higher clinical demands. As simulation complexity increased from level 1 to 3, time to completion of key NR steps decreased non-significantly. Median time to infant drying decreased by 7.5 s (*p* = 0.12), time to placing the infant on the warmer decreased by 21.7 s (*p* = 0.27), and time to the initiation of positive pressure ventilation decreased by 20.8 s (*p* = 0.12). Nevertheless, there remains a need for improvement in absolute time elapsed between delivery and completion of key NR tasks.

**Conclusions:**

PRONTO simulation training enabled nurses in Bihar to maintain core NR skills in simulation despite demands for higher-level triage and management. Although further evaluation of the PRONTO methodology is necessary to understand the full scope of its impact, this analysis highlights the importance of conducting and evaluating simulation training in low-resource settings based on simultaneous care of the mother-infant dyad.

## Background

Between 1980 and 2015 the global early neonatal mortality rate, defined as death within the first 6 days of life, almost halved. However, the percentage of under-five mortality attributable to early neonatal mortality increased from approximately 25 to 35% [1]. In developing regions, neonatal deaths (within the first 27 days of life) accounted for 45% of under-five deaths in 2015 [2]. Within the neonatal period, the largest cause of death globally in 2015 was preterm birth followed by perinatal asphyxia [3]. In the early neonatal period, perinatal asphyxia accounts for over one-fourth of deaths [4]. These statistics underscore the importance of continued global attention to and improvement in immediate newborn care and neonatal resuscitation (NR).

In India the neonatal mortality rate was 29.1 per 1000 live births in 2015 [1]. This was similar to the rate in South Asia as a whole (29.8) and comparable to that of sub-Saharan Africa (27.8), but much higher than the global rate (18.6) [1]. Moreover, India had the highest absolute annual number of neonatal deaths in the world in 2012 at approximately 779,000, a figure that is almost triple the total in Nigeria, the country with the second highest absolute number of neonatal deaths [4].

Bihar is a state in eastern India with a population of 104 million and a 25% population growth rate over the last decade [5]. According to the 2016 Oxford Multidimensional Poverty Index, Bihar is the poorest region in all of South Asia [6]. The neonatal mortality rate in Bihar was 32.2 per 1000 live births in 2012 [7], with significant under-reporting likely. Moreover, the odds of neonatal death in Bihar were reported to more than double with intrapartum maternal complications, including prolonged labor, postpartum hemorrhage, and convulsions, when compared to births without maternal complications (OR 2.17, 95% CI 1.65–2.86) [7]. This underscores the inseparability of neonatal and maternal care at the time of birth in Bihar and similar settings.

Primary health centers (PHCs) in Bihar provide the first point of medical contact with each serving, on average, a rural population of 49,000 [8]. Obstetric and pediatric specialists are very rarely available at PHCs. Such specialists work at referral centers, which in Bihar serve an average catchment area of over 1 million people each [8]. The majority of labor and delivery care in PHCs is provided by nurses with an Auxiliary Nurse and Midwifery (ANM) qualification and occasionally a General Nursing and Midwifery (GNM) qualification, a 2 year and 3.5 year qualification obtained after completion of secondary school respectively [9]. Each PHC often only has one ANM or GNM on duty at any given time [8], which creates challenges for deliveries with multiple complications.

Simulation training is an increasingly utilized methodology for training healthcare providers to manage medical complications such as birth asphyxia. Many NR simulation curriculums have been developed for high-income settings, including the Neonatal Resuscitation Program (NRP) developed by the American Academy of Pediatrics (AAP) [10], the Newborn Life Support course developed by the European Resuscitation Council [11], and the course on Advanced Resuscitation of the Newborn Infant developed by the United Kingdom Resuscitation Council [12]. However, low-resource settings such as Bihar, where providers have little training and are few in number, present unique demands to any simulation-training program. The AAP, World Health Organization, United States Agency for International Development, and other global organizations developed Helping Babies Breathe (HBB) specifically for use in low-resource settings [13].

Both NRP and HBB have been implemented in low-resource settings with mixed results. Studies evaluating pre-training and post-training test scores uniformly found an increase in knowledge after NRP [14–17] and HBB [18–20]. One study also demonstrated that there was no significant loss of knowledge at 6 months post HBB training [20]. However, studies evaluating acquisition of clinical skills after NRP and HBB have reported variable success. After NRP, the percentage of learners that passed a simulation exam varied widely from 14% [17] to 98.6% [14]. Similarly, studies evaluating HBB have demonstrated conflicting results on the presence [20, 21] or lack [19] of improved general performance in observed NR simulations, although improved performance was not sustained at 6 months post-training [20]. Improvement in specific NR skills was equally variable. Studies have reported both an increase in frequency of stimulation and suctioning post-training [22, 23] and a decrease in stimulation with no change in suctioning [21]. Finally, studies have reported an increase in frequency of positive pressure ventilation (PPV) [20], a decrease [22, 23], and no change in PPV skills [19, 21].

PRONTO International has developed a unique approach to NR training in low-resource settings that aims to overcome this knowledge-skill gap. There are five unique components of the PRONTO training. First, the PRONTO training teaches providers to simultaneously manage maternal and neonatal complications rather than focusing on NR skills in isolation. Second, PRONTO simulations are conducted in resource-limited facilities where providers actually work, rather than in classrooms. Third, simulations are high fidelity and include a maternal actor wearing PartoPants™, a hybrid birth simulator, and a NeoNatalie® infant mannequin. Fourth, the PRONTO training implemented in Bihar followed an extended mentorship model spanning 8 months on average. Finally, in addition to evidence-based clinical practices, the PRONTO methodology emphasizes teamwork, compassionate communication with the mother, and structured communication between providers [24].

An evaluation of the PRONTO training methodology in Mexico, implemented in a lower dose modular fashion (3 days) rather than the higher dose mentorship model in Bihar, observed improvements in knowledge related to neonatal and obstetric emergencies as well as improvements in provider self-efficacy and teamwork skills. Additionally, the evaluation observed a reduction in neonatal mortality at 8 months post-training [25, 26]. The impact of the PRONTO strategy on clinical skills and the sustainability of PRONTO’s impact on neonatal mortality is undergoing further evaluation.

Addressing the high neonatal mortality rate in low-resources settings such as Bihar, where providers are scarce [8] and neonatal mortality and maternal intrapartum complications are linked [7], requires caring for mother and infant as a dyad rather than in isolation [27]. The unique focus of the PRONTO training methodology on simultaneous management of mother and infant offers one possible solution.

## Methods

### Aim

The aim of this analysis is to assess the impact of PRONTO simulation training on ANMs/GNMs’ NR skills in Bihar. The specific objective is to assess ANMs/GNMs’ ability to perform core NR skills across simulated scenarios of varying levels of clinical complexity. This objective seeks to evaluate the unique focus of PRONTO simulation training on simultaneous care of the mother-infant dyad.

### Study population

**Fig. 1:**
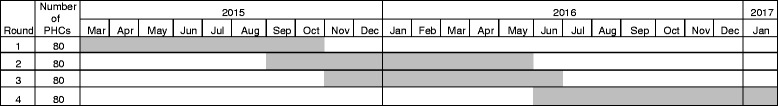
Timeline for Implementation of PRONTO Simulation Training in Bihar

The findings in this manuscript are based on data generated in Phase 1 of the implementation of PRONTO simulation training in Bihar. A total of 658 ANMs/GNMs from 80 PHCs across Bihar participated in Phase 1, which was conducted between March and October 2015. Approximately 89% of nurses in Phase 1 were ANMs and 11% were GNMs. The larger implementation of PRONTO in Bihar included four consecutive phases conducted between August 2015 and January 2017 (Fig. 1). Each phase covered 80 different PHCs across Bihar.

### AMANAT intervention

CARE India [28], in collaboration with the Government of Bihar, implemented a multi-faceted, multi-stakeholder quality improvement project called AMANAT [29], with the goal of decreasing maternal and neonatal mortality in the state. PRONTO International partnered with CARE India to incorporate management of obstetric and neonatal emergencies into the AMANAT intervention using simulation and teamwork training. Mentoring is a core component of the AMANAT intervention. AMANAT Indian nurse mentors possessed a bachelor’s degree in nursing and received a 4-week comprehensive training conducted by CARE India and the Government of Bihar. PRONTO International provided 1 week of training for these mentors on simulation facilitation, team building, communication skills, and debriefing skills. Nurse mentors were then responsible for implementing PRONTO’s curricular components to teach ANM and GNM mentees at PHCs across Bihar.

### PRONTO simulation training

Nurse mentors worked in pairs to facilitate simulation training. Each pair was assigned to 4 PHCs with 6–8 ANM/GNM mentees per PHC. For 8 months, the mentor pairs rotated between their 4 assigned PHCs, spending 1 week of the month at each PHC. The PRONTO-specific curriculum focused on team building in week 1, normal spontaneous vaginal delivery (NSVD) and immediate newborn care in week 2, NR and post-partum hemorrhage (PPH) in week 3, and repeated simulations of NSVD, NR, and PPH along with other obstetric and neonatal emergencies in the remaining weeks. Throughout the training period, nurse mentors could conduct additional simulations, complementary case-based learning, and skills stations, as they deemed appropriate.

A total of 9 different PRONTO NR simulations are included in this analysis. To be included, the simulation had to require resuscitation of a non-vigorous infant with progression to PPV according to the AAP NR algorithm [30], which is consistent with Indian guidelines [31]. Simulations were of varying levels of clinical complexity. Level 1 simulations required only resuscitation of a non-vigorous infant without maternal complications. Level 2 simulations required NR and attention to maternal complications, which were sequential, with the maternal complication having been resolved before the need for NR (i.e. shoulder dystocia). Level 3 simulations were those in which the infant required resuscitation at the same time as the mother experienced a complication, thus requiring higher-level triage and management (i.e. immediate PPH). After every simulation, a video-guided debrief was conducted focusing on “what went well,” “what needed improvement,” and “what could be done differently when faced with a similar scenario in the future.”

### Simulation video monitoring

All simulations were video-recorded as part of the training protocol to allow mentees to learn from their own performance during simulation debriefing. After the simulation session, videos were transferred to encrypted USB drives and transported to Patna, the capital of Bihar, where they were uploaded to an encrypted server. For each PHC, one simulation video from each category, including NSVD, NR, and PPH, was coded for pre-defined clinical skills as well as teamwork and communication skills at weeks 3, 5, and 7 of the mentorship program. If the simulation video for a specified week was missing due to issues such as file transport, video recording error, or delays in mentoring due to holidays or natural disasters, a corresponding video from the prior week of mentorship was used. If that was not available, then the simulation video from the week after was used.

**Fig. 2:**
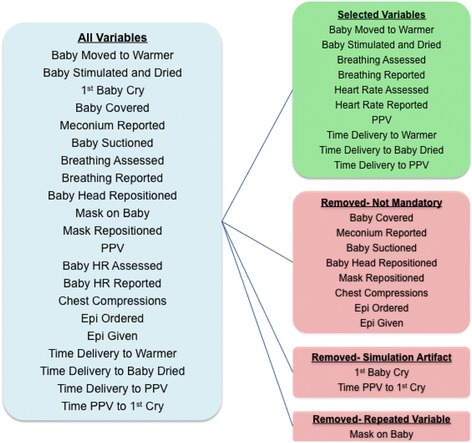
Final Variable Selection and Reason for Variable Removal

Codes for video analysis were chosen by a team of clinical and simulation training experts. Not all codes are included in this analysis. Clinical skills that were not mandatory in every NR simulation (i.e., ‘meconium reported’) were dropped, as were codes that repeated another variable and codes thought to represent simulation artifact (i.e., ‘baby cried’ was a decision made by simulation facilitators rather than a reflection of mentees’ skills) (Fig. 2 in Appendix). Simulation video coding was completed by two local Hindi-speaking nurses using Studiocode™ software [32]. Fifteen percent of videos were randomly selected for double coding by the video analyst team. The inter-rater reliability of the double-coded videos was 93% for clinical skill codes and 81% for teamwork and communications codes.

Both mentors and mentees provided written informed consent for use of the video data in an aggregated analysis. Ethical approval was granted from the Committee on Human Research at the University of California San Francisco (14–15,446) and the Institutional Review Board of the Indian Institute of Health Management Research.

### Statistical analysis

To achieve the stated objective and capture the unique emphasis of PRONTO training on care of the mother-infant dyad, mentees’ NR skills were compared across the pre-defined levels of simulation complexity. A Pearson’s chi-square test was used to compare the percent of simulations in which key NR steps were completed by level of simulation complexity. If the expected cell count assumption was not met, Fisher’s exact test was substituted. A Mann Whitney-U test of medians was used to compare time to completion of key steps in the NR algorithm by level of simulation complexity due to violation of the normality assumption of parametric methods.

A second analysis was conducted using stratified data to understand the effectiveness of the PRONTO methodology in teaching mentees to provide newborn clinical care despite the reality of provider shortages and competing priorities. Data were stratified by number of participants in a given simulation, and simulations with only 1–2 mentees present were analyzed using the same statistical methods.

**Fig. 3:**
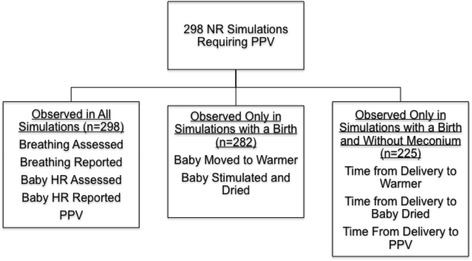
Sample Size for Selected Variables

All level 2 simulations were meconium deliveries, which were excluded from time-based analyses because they required an extra step for suctioning. Therefore the evaluation of time-based variables was limited to a comparison of complexity levels 1 and 3. To be consistent, all analyses were limited to a comparison of levels 1 and 3. However, percent completion of key NR steps was reported for level 2. Since 9 simulated NR scenarios with different observable steps are included in this analysis, there is variation in the sample size for each variable. Sample size is reported for each variable in every table and further explained in Fig. 3 in Appendix. All statistical analyses were conducted in IBM SPSS Statistics 23 [33].

## Results

A total of 513 simulation videos were analyzed with Studiocode™ from Phase 1 of PRONTO training. Of these, 298 required NR with progression to PPV. The distribution of these simulations according to week of training and level of complexity is described in Table 1. In Phase 1, 11.4% of NR simulations conducted were level 1, 12.8% were level 2, and 75.8% were level 3. Approximately half of level 1 simulations were conducted during training weeks 2–3, and approximately half of level 3 simulations were conducted during training weeks 6–7.

**Table 1:** Simulation Difficulty Across Time

	Level of Clinical Complexity			
	Level 1	Level 2	Level 3	Total
Week of Training	n (column %)			
Week 2–3	16 (47.1)	25 (65.8)	17 (7.5)	58
Week 4–5	11 (32.4)	11 (28.9)	86 (38.1)	108
Week 6–7	7 (20.6)	2 (5.3)	123 (54.4)	132
Total	34	38	226	298

### Essential newborn care and resuscitation of a non-vigorous infant

As the complexity of simulations increased from level 1, requiring care of a non-vigorous infant only, to level 3, requiring simultaneous management of maternal and neonatal emergencies, the percentage of simulations in which mentees completed key steps of NR did not change significantly (Table 2). As clinical complexity increased, the percentage of simulations in which the non-vigorous infant was moved to a warmer remained above 86%, the percentage of simulations in which the infant was dried and stimulated remained above 84%, and the percentage of simulations in which the infant received PPV remained above 89%. Assessment and communication of respiratory status were consistently low. Assessment of respiratory rate ranged from 5.9% in level 1 simulations to 8.4% in level 3 simulations. Communication of respiratory rate ranged from 5.9% in level 1 simulations to 8.8% in level 3 simulations.

**Table 2:** Completion of Key Steps of Newborn Care and NR by Level of Simulation Complexity

		Level 1	Level 2	Level 3	
Key NR Step	n^a^	%^b^			*p*-value
Baby moved to warmer	282	94.4	92.1	86.7	0.48^c^
Baby stimulated and dried	282	94.4	84.2	88.9	0.70^c^
Breathing assessed	298	5.9	10.5	8.4	1.00^c^
Breathing reported	298	5.9	10.5	8.8	0.75^c^
Heart rate assessed	298	79.4	78.9	69.0	0.22^d^
Heat rate reported	298	50.0	60.5	50.0	1.00^d^
PPV	298	94.1	89.5	90.3	0.75^c^

*NR* neonatal resuscitation, *PPV* positive pressure ventilation

^a^Total number of simulations with step observable, including all levels of complexity

^b^Percent of simulations in which step was completed

^c^Fisher’s exact test comparing Level 1 vs. Level 3

^d^Pearson’s chi-square test comparing Level 1 vs. Level 3

As complexity of simulations increased from level 1 to level 3, the time elapsed between delivery and key NR steps decreased, although not significantly. The median time from delivery to infant drying was 7.5 s faster in level 3 simulations compared to level 1 simulations (*p* = 0.12). The median time from delivery to placing the infant in the warmer was 21.7 s faster in level 3 simulations (*p* = 0.27). The median time from delivery to initiation of PPV was 20.8 s faster in level 3 simulations (*p* = 0.12) (Table 3).

**Table 3:** Time to Completion of Key Steps of Newborn Care and NR by Level of Simulation Complexity

		Level 1	Level 3		
Key NR Step	n^a^	Median in Seconds (Interquartile Range)		Difference^b^	*p*-value^c^
Time- delivery to baby dried	225	19.3 (6.4–65.3)	11.8 (6.7–24.8)	−7.5	0.12
Time- delivery to warmer	225	72.3 (29.3–93.8)	50.6 (32.7–71.6)	−21.7	0.27
Time- delivery to PPV	225	112.8 (82.4–142.7)	92.0 (65.8–121.1)	−20.8	0.12

*NR* neonatal resuscitation, *PPV* positive pressure ventilation

^a^Total number of simulations with step observable, including all levels of complexity

^b^Difference in seconds between Level 3 and Level 1

^c^Mann Whitney-U Test comparing Level 1 vs. Level 3

### Clinical scenarios with a limited number of providers

Approximately half of the simulations requiring NR with progression to PPV had 1–2 participants, and the other half had 3–4 participants. This was true regardless of the level of clinical complexity (47.1% of level 3 simulations had 1–2 participants).

When simulations were conducted with only 1–2 participants, the percentage of simulations in which mentees completed key steps of NR did not change significantly as simulation complexity increased to level 3 (Table 4). Additionally, despite having fewer participants, the change in time to completion of key NR steps suggested these steps were completed more quickly with increasing clinical complexity. The median time from delivery to baby dried was 8.4 s faster in level 3 simulations compared to level 1 simulations (*p* = 0.17). The median time from delivery to initiation of PPV was 20.8 s faster in level 3 simulations (*p* = 0.10) (Table 5).

**Table 4:** Completion of Key Steps of Newborn Care and NR in Simulations with 1–2 Participants

		Level 1	Level 2	Level 3	
Key NR Step	n^a^	%^b^			*p*-value
Baby moved to warmer	130	100.0	89.5	92.9	1.00^c^
Baby stimulated and dried	130	100.0	84.2	96.0	1.00^c^
Breathing assessed	130	0.0	5.3	3.0	1.00^c^
Breathing reported	130	0.0	5.3	4.0	1.00^c^
Heart rate assessed	130	91.7	78.9	71.7	0.18^c^
Heart rate reported	130	50.0	63.2	45.5	0.77^d^
PPV	130	100.0	94.7	94.9	1.00^c^

*NR* neonatal resuscitation, *PPV* positive pressure ventilation

^a^Total number of simulations with step observable, including all levels of complexity

^b^Percent of simulations in which step was completed

^c^Fisher’s exact test comparing Level 1 vs. Level 3

^d^Pearson’s chi-square test comparing Level 1 vs. Level 3

**Table 5:** Time to Completion of Key Steps of Newborn Care and NR in Simulations with 1–2 Participants

		Level 1	Level 3		
Key NR Step	n^a^	Median in Seconds (Interquartile Range)		Difference^b^	*p*-value^c^
Time- delivery to baby dried	108	21.6 (9.9–60.5)	13.2 (7.6–29.1)	−8.4	0.17
Time- delivery to warmer	108	40.5 (28.5–116.7)	51.1 (35.8–70.0)	10.6	0.97
Time- delivery to PPV	108	112.8 (85.0–155.6)	92.0 (68.0–114.7)	−20.8	0.10

*NR* neonatal resuscitation, *PPV* positive pressure ventilation

^a^Total number of simulations with step observable, including all levels of complexity

^b^Difference in seconds between Level 3 and Level 1

^c^Mann Whitney-U Test comparing Level 1 vs. Level 3

## Discussion

The PRONTO International training program is the only simulation-training program that teaches simultaneous management of obstetric and neonatal emergencies and is contextually designed for low-resource settings. Moreover, this analysis is the first evaluation of a NR simulation-training program that measures clinical performance in simulation by complexity of the simulated clinical scenario. This type of evaluation is critically important in extremely resource-limited settings, such as Bihar, where providers are scarce and must constantly juggle competing demands for their time and attention, an important barrier to the provision of optimal care [8].

Over the course of PRONTO training, ANM and GNM mentees in Bihar were presented with clinical scenarios of increasing complexity with half of the level 3 scenarios occurring during the final weeks of training. As complexity of the simulated scenarios increased, requiring simultaneous management of maternal and neonatal complications, the percentage of simulations in which mentees completed key steps of NR did not change. This suggests that the PRONTO training successfully enabled ANMs and GNMs to maintain NR skills despite increasing demands for triage and high-level management. This was true even with only 1–2 mentees attending to both mother and infant during a complicated delivery, a frequent occurrence in Bihar. Additionally, as the complexity of the clinical scenarios increased, the data suggest that mentees completed key steps in NR with increased efficiency, even with only 1–2 participants present. Although not statistically significant, these decreases are clinically significant given the poor baseline level of NR skills in Bihar. This increase in efficiency is likely related to increasing skill and knowledge over the timeframe of the intervention, as more level 3 simulations occurred later.

Nonetheless, the data clearly demonstrate the need for further improvement of clinical skills in Bihar. Urgency is one key area for improvement. The median time elapsed between delivery and the initiation of PPV was more than triple the evidence-based guideline of 30 s [30]. Additionally, rates of measuring and reporting respiratory status were much lower than the completion rate of other core skills reported in Tables 2 and 4. Measurement of respiratory rate may be artificially low as respiratory status can be assessed without a distinct action and thus may have been under-reported by video analysts. The low rate of reporting respiratory status as well as heart rate, a key skill in NR that requires verbalizing this information, may also suggest a component of inexperience with teamwork and communication in mentees that are used to working alone.

The results from this analysis are consistent with existing literature evaluating the impact of simulation training on clinical skill acquisition, which demonstrates variable results [14, 16, 19–23]. This analysis adds to the existing body of evidence because of the unique complexity-based approach that it utilizes to evaluate the PRONTO methodology. Another strength and contribution of this analysis is the large amount of high quality data provided by the video-recorded simulations. This is rare in low-resource settings and offers tremendous opportunity for further analysis.

Limitations of the analysis include inability to assess the quality of clinical skills, such as presence or absence of chest rise during PPV. Also, this analysis does not directly assess the change in mentees’ skills over time. Finally, this analysis does not address mentees’ ability to manage maternal complications when neonatal complications occur simultaneously. This is important given PRONTO’s focus on the mother-infant dyad and will be addressed, along with the other limitations of scope, in analyses of the subsequent three phases of PRONTO training in Bihar.

Further evaluation is also required to investigate of the impact of the PRONTO training on clinical outcomes in real deliveries. Existing NR simulation literature demonstrates that translation of knowledge gained during simulation into improved clinical outcomes in real births is difficult and variable. After both NRP and HBB, studies reported no significant change in all-cause early neonatal mortality, stillbirth, and perinatal mortality [34–37]. Additionally, there have been reports demonstrating both a decrease [22, 23] and a lack of effect [37] on neonatal mortality at 24 h. Moreover, studies have reported a decrease in mortality pre-discharge [14], mortality due to birth asphyxia [38], and stillbirth rates [22]. It is our hope that the unique features of the PRONTO training in Bihar including long duration of teaching, in-situ simulation, attention to teamwork and communication skills, and focus on simultaneous management of the mother-infant dyad create a training environment that replicates the clinical environment of low-resource settings, and thus facilitates translation of skills learned during simulations to real clinical scenarios. In combination with other components of the AMANAT program, we hope that the translation of skills learned in PRONTO simulation training will contribute to improved neonatal and maternal survival in Bihar.

## Conclusion

PRONTO simulation training enabled ANMs and GNMs in Bihar to maintain core NR skills in simulation as clinical complexity increased from isolated management of an asphyxiated infant to simultaneous management of birth asphyxia and a maternal complication, such as PPH. Additionally, as the clinical complexity of simulated scenarios increased, mentees were able to complete key steps of NR more quickly. Although these decreases in time to completion of a key task are not statistically significant, they are clinically important in Bihar, the poorest region in South Asia. Further evaluation of the PRONTO training is necessary to fully assess its impact on maternal and neonatal health outcomes in Bihar. This analysis, however, highlights the importance of conducting and evaluating simulation training based on the mother-infant dyad in low resource settings, where care of mothers and infants is rarely conducted in isolation.

## Appendix

### PRONTO Phase 1 Neonatal Resuscitation Simulation Data

The appendix provides an illustration of both the selection process for variables in Tables 2, 3, 4 and 5 (Fig. 2) and sample sizes for each variable (Fig. 3).

## Abbreviations

AAP: American Academy of Pediatrics
ANM: Auxiliary nurse midwifery
CI: Confidence interval
GNM: General nursing and midwifery
HBB: Helping Babies Breathe
NR: Neonatal resuscitation
NRP: Neonatal Resuscitation Program
NSVD: Normal spontaneous vaginal delivery
PHC: Primary health center
PPH: Post-partum hemorrhage
PPV: Positive pressure ventilation

## References

[1] GBD 2015 Child Mortality Collaborators (2016). Global, regional, national, and selected subnational levels of stillbirths, neonatal, infant, and under-5 mortality, 1980-2015: a systematic analysis for the global burden of disease study 2015. Lancet.

[2] You D, Hug L, Ejdemyr S, Beise J. Levels & Trends in child mortality report 2015: UNICEF; 2015. http://www.who.int/maternal_child_adolescent/documents/levels_trends_child_mortality_2015/en/. Accessed 20 Feb 2016.

[3] Liu L, Oza S, Hogan D, Chu Y, Perin J, Zhu J, Lawn JE, Cousens S, Mathers C, Black RE (2016). Global, regional, and national causes of under-5 mortality in 2000-15: an updated systematic analysis with implications for the sustainable development goals. Lancet.

[4] Lawn JE, Blencowe H, Oza S, You D, Lee AC, Waiswa P (2014). Every newborn: progress, priorities, and potential beyond survival. Lancet.

[5] Office of the director of census operations (2011). Census of India 2011: provisional population totals.

[6] Multidimensional Poverty Index 2016 Highlights ~ South Asia (2016). Oxford poverty and human development index.

[7] Kumar GA, Dandona R, Chaman P, Singh P, Dandona L (2014). A population-based study of neonatal mortality and maternal care utilization in the Indian state of Bihar. BMC Pregnancy Childbirth.

[8] Sharma BP. Rural Health Statistics: Government of India Ministry of Health and Family Welfare Statistics Division; 2015. http://www.indiaenvironmentportal.org.in/files/file/Rural%20Health%20Statistics%202014-15.pdf. Accessed 20 Feb 2016

[9] Types of Nursing Programs. Indian nursing council. http://www.indiannursingcouncil.org/nursing-programs.asp?show=prog-type. Accessed 2 June 2017.

[10] Neonatal Resuscitation Program. https://www.aap.org/en-us/continuing-medical-education/life-support/NRP/Pages/NRP.aspx. Accessed 20 Feb 2016.

[11] European Resuscitation Council: Newborn life support. https://www.erc.edu/courses/newborn-life-support. Accessed 9 Mar 2017.

[12] Resuscitation Council UK: Advanced resuscitation of the newborn infant. https://www.resus.org.uk/information-on-courses/advanced-resuscitation-of-the-newborn-infant/. Accessed 9 Mar 2017.

[13] Helping Babies Breathe. http://www.helpingbabiesbreathe.org Accessed 20 Feb 2016.

[14] Charafeddine L, Badran M, Nakad P, Ammar W, Yunis K. Strategic assessment of implementing neonatal resuscitation training at a National Level. Pediatr Int. 2015;58(7):595–600. 10.1111/ped.12868 26662795

[15] Xu T, Wang H, Gong L, Ye H, Yu R, Wang D (2014). The impact of an intervention package promoting effective neonatal resuscitation training in rural China. Resuscitation.

[16] Lai NM, Ngim CF, Fullerton PD (2012). Teaching medical students neonatal resuscitation: knowledge gained and retained from a brief simulation-based training workshop. Educ Health (Abingdon).

[17] Jabir MM, Doglioni N, Fadhil T, Zanardo V, Trevisanuto D (2009). Knowledge and practical performance gained by Iraqi residents after participation to a neonatal resuscitation program course. Acta Paediatr.

[18] Seto TL, Tabangin ME, Josyula S, Taylor KK, Vasquez JC, Kamath-Rayne BD (2015). Educational outcomes of helping babies breathe training at a community hospital in Honduras. Perspect Med Educ.

[19] Singhal N, Lockyer J, Fidler H, Keenan W, Little G, Bucher S (2012). Helping babies breathe: global neonatal resuscitation program development and formative educational evaluation. Resuscitation.

[20] Bang A, Patel A, Bellad R, Gisore P, Goudar SS, Esamai F (2016). Helping babies breathe (HBB) training: what happens to knowledge and skills over time?. BMC Pregnancy Childbirth.

[21] Ersdal HL, Vossius C, Bayo E, Mduma E, Perlman J, Lippert A (2013). A one-day “helping babies breathe” course improves simulated performance but not clinical management of neonates. Resuscitation.

[22] Msemo G, Massawe A, Mmbando D, Rusibamayila N, Manji K, Kidanto HL (2013). Newborn mortality and fresh stillbirth rates in Tanzania after helping babies breathe training. Pediatrics.

[23] Mduma E, Ersdal H, Svensen E, Kidanto H, Auestad B, Perlman J (2015). Frequent brief on-site simulation training and reduction in 24-h neonatal mortality--an educational intervention study. Resuscitation.

[24] PRONTO International. http://prontointernational.org. Accessed 20 Feb 2016.

[25] Walker D, Cohen S, Fritz J, Olvera M, Lamadrid-Figueroa H, Cowan JG (2014). Team training in obstetric and neonatal emergencies using highly realistic simulation in Mexico: impact on process indicators. BMC Pregnancy Childbirth.

[26] Walker DM, Cohen SR, Fritz J, Olvera-GarcÌa M, Zelek ST, Fahey JO, et al. Impact evaluation of PRONTO Mexico: a simulation-based program in obstetric and neonatal emergencies and team training. Simul Healthc 2016;11(1):1-9. 10.1097/SIH.0000000000000106 PMC5367503 26312613

[27] Starrs AM (2014). Survival convergence: bringing maternal and newborn health together for 2015 and beyond. Lancet.

[28] CARE India. https://www.careindia.org. Accessed 20 Feb 2016.

[29] Das A, Nawal D, Singh MK, Karthick M, Pahwa P, Shah MB (2016). Impact of a nursing skill-improvement intervention on newborn-specific delivery practices: an experience from Bihar, India. Birth.

[30] Weiner G, Zaichkin J (2016). Textbook of neonatal resuscitation.

[31] Gupta P, Committee IL (2000). Guidelines 2000 for neonatal resuscitation. Indian Pediatr.

[32] Studiocode Group. http://www.studiocodegroup.com/. Accessed 20 Feb 2016.

[33] IBM Corp. Released 2013IBM SPSS statistics for Macintosh, version 23.0. Armonk: IBM Corp.

[34] Goudar SS, Dhaded SM, McClure EM, Derman RJ, Patil VD, Mahantshetti NS (2012). ENC training reduces perinatal mortality in Karnataka, India. J Matern Fetal Neonatal Med.

[35] Carlo WA, Goudar SS, Jehan I, Chomba E, Tshefu A, Garces A (2010). High mortality rates for very low birth weight infants in developing countries despite training. Pediatrics.

[36] Carlo WA, Goudar SS, Jehan I, Chomba E, Tshefu A, Garces A (2010). Newborn-care training and perinatal mortality in developing countries. N Engl J Med.

[37] Bellad RM, Bang A, Carlo WA, McClure EM, Meleth S, Goco N (2016). A pre-post study of a multi-country scale up of resuscitation training of facility birth attendants: does helping babies breathe training save lives?. BMC Pregnancy Childbirth.

[38] Deorari AK, Paul VK, Singh M, Vidyasagar D, Network MC (2001). Impact of education and training on neonatal resuscitation practices in 14 teaching hospitals in India. Ann Trop Paediatr.

